# Oxylipin Diversity in the Diatom Family Leptocylindraceae Reveals DHA Derivatives in Marine Diatoms

**DOI:** 10.3390/md12010368

**Published:** 2014-01-17

**Authors:** Deepak Nanjappa, Giuliana d’Ippolito, Carmela Gallo, Adriana Zingone, Angelo Fontana

**Affiliations:** 1 Stazione Zoologica Anton Dohrn, Villa Comunale, Naples 80121, Italy; E-Mails: depac.n@gmail.com (D.N.); zingone@szn.it (A.Z.); 2 CNR-Institute Biomolecular Chemistry, Via Campi Flegrei 34, Pozzuoli (Naples) 80078, Italy; E-Mails: carmen.gallo@icb.cnr.it (C.G.); angelo.fontana@icb.cnr.it (A.F.)

**Keywords:** oxylipins, diatoms, *Leptocylindrus*, lipoxygenase pathways, eicosapentaenoic acid, docosahexaenoic acid

## Abstract

Marine planktonic organisms, such as diatoms, are prospective sources of novel bioactive metabolites. Oxygenated derivatives of fatty acids, generally referred to as oxylipins, in diatoms comprise a highly diverse and complex family of secondary metabolites. These molecules have recently been implicated in several biological processes including intra- and inter-cellular signaling as well as in defense against biotic stressors and grazers. Here, we analyze the production and diversity of C_20_ and C_22_ non-volatile oxylipins in five species of the family Leptocylindraceae, which constitute a basal clade in the diatom phylogeny. We report the presence of species-specific lipoxygenase activity and oxylipin patterns, providing the first demonstration of enzymatic production of docosahexaenoic acid derivatives in marine diatoms. The differences observed in lipoxygenase pathways among the species investigated broadly reflected the relationships observed with phylogenetic markers, thus providing functional support to the taxonomic diversity of the individual species.

## 1. Introduction

Diatoms are unicellular photosynthetic organisms that play a pivotal role in the Earth’s ecosystem, where they are deemed to contribute to *ca.* 40% of the 45–50 billion metric tons of the organic carbon fixed annually in the oceans [1]. These highly diverse microalgae are found worldwide and populate almost all the aquatic ecosystem with adequate light and nutrients, thriving both in benthic and in planktonic environments. It is estimated that around 100,000 diatom species exist, of which approximately only 12,000 currently described [2,3,4]. This discrepancy is explained with the relatively low number of taxa so far cultivated and examined with modern techniques, as well as with the fact that wide parts of the aquatic environments are still unexplored [4].

In recent years, a combination of different methods, involving microscopy and molecular markers (DNA sequences) have been applied to distinguish and identify diatom species, which have led to the evidence that many species, defined as cryptic or pseudocryptic, are hardly distinguishable morphologically. Cryptic diversity has been well documented in the pennate diatoms, for example the planktonic genus *Pseudo-nitzschia* [5,6,7,8] and the benthic genus *Sellaphora* [9,10,11,12], while the recent discoveries in centric diatoms, for example *Skeletonema costatum* sensu lato [13,14,15] and *Leptocylindrus danicus* sensu lato [16] have further demonstrated that diatom diversity is currently underestimated. The description of almost undistinguishable species on the other hand has raised debates on their actual functional diversity, which calls for knowledge on biochemical, physiological, and ecological differences among cryptic species. Though there have been few studies in this direction, evidence is accumulating that morphologically similar species do differ in physiological characteristics, which can have profound influence on their ecological requirements and functional roles [17,18,19,20,21].

Marine diatoms produce a plethora of ecological and physiological mediators, called oxylipins, which are oxygenated compounds biosynthesized from polyunsaturated fatty acids (PUFAs) [22]. Oxylipin biosynthesis involves, as the first committed step, the oxidation of fatty acids to hydroperoxides by LipOXygenases (LOXs), non-heme iron dioxygenases that add molecular oxygen to the carbon chain of fatty acids and are classified according to the specificity of oxygen position. In plants oxygenation typically occurs at C-9 or C-13 of C_18_ chains of linoleic and linolenic acids, while in diatoms LOX activities generally involve C_16_ and C_20_ PUFAs [23] with highly diverse position of oxygen insertion that apparently is associated to species-specificity [24,25]. The attention of most researchers has focused on the volatile compounds produced by hydroperoxyde lyases (HPL) products such as 2,4 decadienal, while the physiological role of non-volatiles oxylipins (NVO) remains less studied [24,25]. Recently, the distribution of these compounds within taxonomically-related diatoms has been proposed as a complementary or alternative tool to classify and identify cryptic species [26]. Among diatoms, Leptocylindraceae constitute an early-diverging branch in the evolutionary tree with a limited number of species that show a high morphological similarity, despite deep differences in their molecular markers [16]. In this study, we used a targeted liquid chromatography-mass spectrometry (LC-MS) profiling method to address the identification and variability of NVOs in five distinct species of *Leptocylindraceae* diatoms collected at the Long Term Ecological Research station MareChiara (LTER-MC), in the Gulf of Naples (GoN, Tyrrhenian Sea, Mediterranean Sea). The aim of this study was to investigate oxylipin production in an ancient diatom group in order to highlight functional differences among pseudocryptic species in the family and test the possibility to use specific metabolites as functional biomarkers.

## 2. Results and Discussion

### 2.1. Oxylipin Characterization

**Figure 1:**
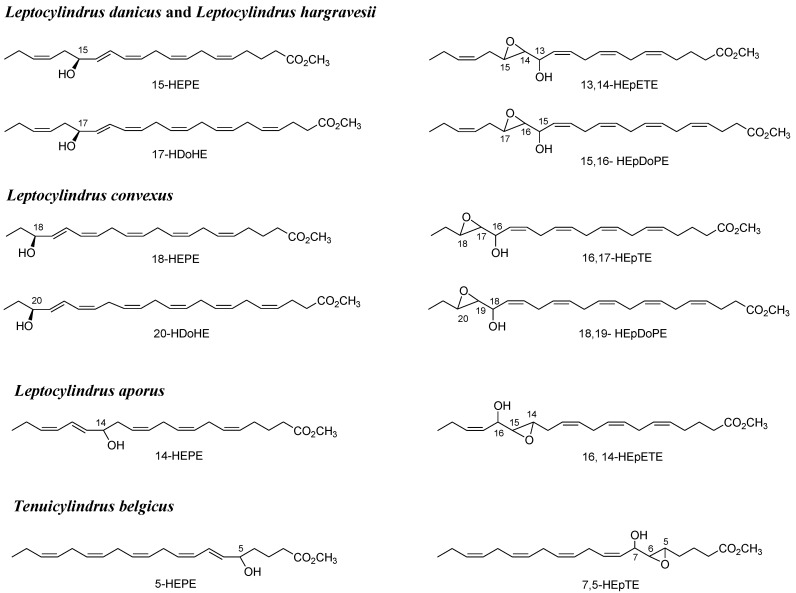
Oxylipins characterized by LC/MS/MS as methyl ester in *Leptocylindraceae* species. 15-hydroxy-eicosa-5,8,11,13,17-pentaenoic acid (15-HEPE); 13-hydroxy-14,15-epoxyeicosa-5,8,11,17-tetraenoic acid (13,14-HEpETE); 17-hydroxy-docosa-4,7,10,13,15,19-hexaenoic acid (17-HDoHE); 15-hydroxy-16,17-epoxy-docosa-4,7,10,13,19-pentaenoic acid (15,16-HEpDoPE); 18-hydroxy-eicosa-5,8,11,14,16-pentaenoic acid (18-HEPE); 16-hydroxy-17,18-epoxy-eicosa-5,8,11,14-tetraenoic acid (16,17-HEpTE); 20-hydroxy-docosa-4,7,10,13,16,18-hexaenoic acid (20-HDoHE); 18-hydroxy-19,20-epoxydocosa-4,7,10,13,16-pentaenoic acid (18,19-HEpDoPE); 14-hydroxy-eicosa-5,8,11,15,17-pentaenoic acid (14-HEPE); 16-hydroxy-14,15-epoxy-eicosa-5,8,11,17-tetraenoic acid (16,14-HEpETE); 5-hydroxy-eicosa-6,8,11,14,17-pentaenoic acid (5-HEPE); 7-hydroxy-5,6-epoxy-eicosa-8,11,14,17-tetraenoic acid (7,5-HEpTE).

**Figure 2:**
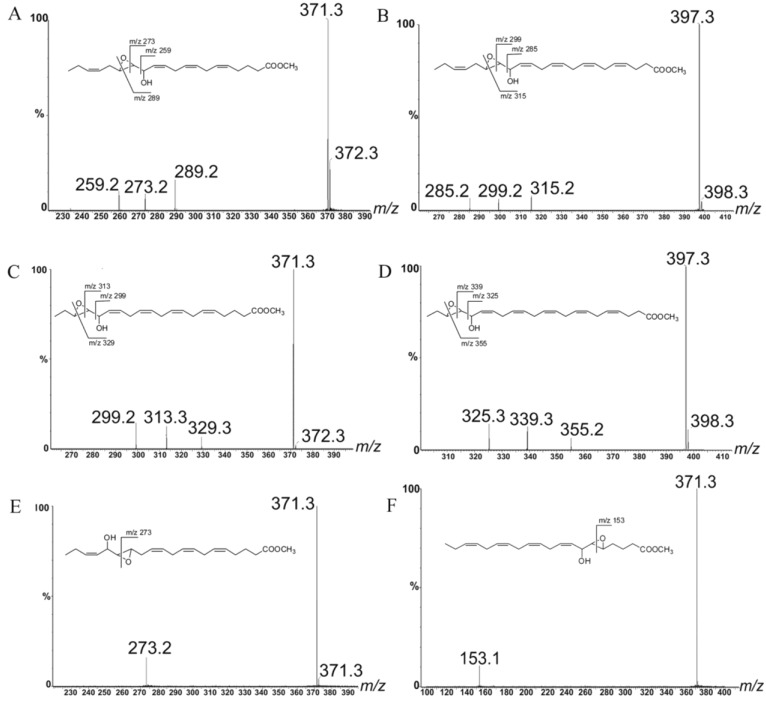
MS/MS spectra and the fragmentation sites of epoxyalcohol derivatives: (**A**) 13,14-HEpETE, and (**B**) 15,16-HEpDoPE of *L. danicus* and *L. hargravesii*; (**C**) 16,17-HEpTE, and (**D**) 18,19-HEpDoPE of *L. convexus*; (**E**) 16,14-HEpETE of *L. aporus*, and (**F**) 7,5-HEpTE of *T. belgicus*.

LOX products were investigated in nine strains of four different *Leptocylindrus* species and two strains of *Tenuicylindrus belgicus* (Supplementary Information, Figure S1) isolated from the GoN. Analyses were carried out by positive Electrospray ionization (ESI) tandem mass spectrometry in agreement with Cutignano *et al.* [27]. With the exception of one strain of *L. aporus* (SZN-B651), all samples under investigation showed LC-MS peaks due to NVOs deriving from either eicosapentaenoic acid (EPA, C20:5 ω3) or docosahexaenoic acid (DHA, C22:6 ω3) (Supplementary Information, Figure S2). These products differed by 26 atomic mass units and were identified as methyl derivatives of hydroxyacid (*m/z* 355; C_21_H_32_NaO_3_^+^) and epoxyalcohol (*m/z* 371; C_21_H_32_NaO_4_^+^) of EPA [27] or hydroxyacid (*m/z* 381; C_23_H_34_NaO_3_^+^) and epoxyalcohol (*m/z* 397; C_23_H_34_NaO_4_^+^) of DHA (Figure 1). As the DHA derivatives have never been described in diatoms, their assignment was supported by the similarity of fragmentation with the EPA derivatives. In fact, in *L. danicus* and *L. hargravesii*, the ion cluster at *m/z* 285, 299 and 315 was assigned to fragmentation of 15-hydroxy-16,17-epoxy-docosa-4,7,10,13,19-pentaenoic acid (15,16-HEpDoPE) (*m/z* 397) on the basis of the homologous MS cluster (*m⁄z* 259, 273 and 289) of 13-hydroxy-14,15-epoxyeicosa-5,8,11,17-tetraenoic acid (13,14-HEpETE) (*m/z* 371) (Figure 2A,B) [27]. Analogously, the products at *m/z* 371 (14.1 min) and 397 (17.4 min) in the spectrum of *L. convexus* were identified as 16-hydroxy-17,18-epoxy-eicosa-5,8,11,14-tetraenoic acid (16,17-HEpTE) and 18-hydroxy-19,20-epoxydocosa-4,7,10,13,16-pentaenoic acid (18,19-HEpDoPE) according to the similarities in their mass spectra (Figure 2C,D). Noteworthy, the co-occurrence of homologous compounds in these three species is in agreement with the mechanistic model on the structural basis for the positional specificity of mammalian LOXs [28,29]. In fact, according to the space-related hypothesis, polyunsaturated fatty acids are docked into the hydrophobic catalytic site of the enzyme with the methyl end extending down into the bottom of the pocket. The binding model explains the homology between EPA and DHA derivatives and why oxidation of these fatty acids takes place in the same position, starting from the methyl end in each *Leptocylindrus* species, as the location of the chain establishes the positional specificity of lipoxygenation. These data also suggest that the same LOX enzymes are responsible for the homologous pairs of NVOs in the diatoms. The other two species, *L. aporus* and *T. belgicus*, showed the prevailing presence of EPA derivatives with insignificant levels of DHA-based NVOs. Tandem MS/MS data of the epoxyalcohol from the former species indicated the occurrence of a single ion at *m/z* 273 as expected for fragmentation of 16-hydroxy-14,15-epoxy-eicosa-5,8,11,17-tetraenoic acid (16,14-HEpETE) (Figure 2E), whereas, the epoxyalcohol of *T. belgicus* gave a unique peak at *m/z* 153, which was consistent with the presence of 7-hydroxy-5,6-epoxy-eicosa-8,11,14,17-tetraenoic acid (7,5-HEpTE) [27].

It is of note that the diatom extracts also contained minor products that did not give diagnostic fragmentation pattern in LC/MS/MS and that are not taken into consideration in this study (Supplementary Information, Figure S2).

**Figure 3:**
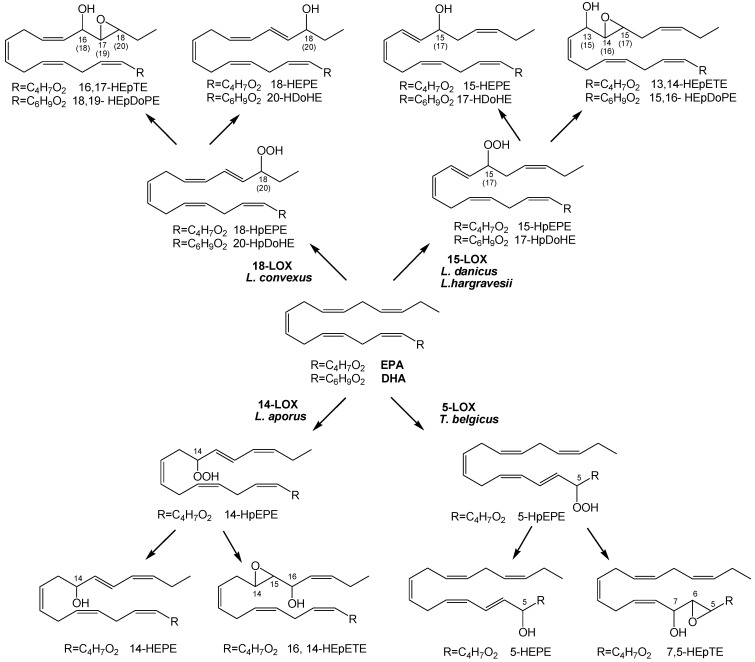
Lipoxygenase pathways in *Leptocylindraceae* species. Relevant carbon atoms for DHA derivatives were indicated in parenthesis.

Epoxyalcohol fragmentation was diagnostic to deduce the position of lipoxygenation in these species. In fact, biosynthesis of the epoxyalcohols implies reduction of the hydroperoxide generated by LOX to the respective alcohol by transferring an oxygen atom to the vicinal double bond. In higher eukaryotes, the reaction is catalyzed by epoxyalcohol synthase (EAS) and proceeds with high stereospecificity [30,31]. As this mechanism establishes a clear correlation between the position of hydroxy and epoxy groups with the site of peroxygenation, the MS/MS characterization of the epoxyalcohols is indicative of LOX oxidation at C-14 of EPA in *L. aporus* (14-LOX), at C-15 of EPA and C-17 of DHA in *L. danicus* and *L. hargravesii* (15-LOX), at C-18 of EPA and C-20 of DHA in *L. convexus* (18-LOX) and, finally at C-5 in *T. belgicus* (5-LOX) (Figure 3).

As far as we know, only LOX derivatives of EPA and C_16_ fatty acids, hexadecatrienoic and hexadecatetraenoic acid [22,25,32,33], have been detected hitherto in diatoms, whereas derivatives of arachidonic acid (AA) and EPA have also been reported in other marine organisms, such as red algae and sponges [34,35]. In this study, oxylipin derivatives of DHA were found for the first time in at least three species of the genus *Leptocylindrus*, thus providing the first finding of these compounds in marine organisms. In mammals, the oxylipins derived from peroxidation of DHA, *i.e.*, the D-series resolvins (neuro-)protectin D1 and maresins, are important compounds with multiple activities as endogenous local mediators, with strong anti-inflammatory effects in addition to some immunoregulatory activities [36,37].

### 2.2. Stereochemistry of Hydroacids

**Figure 4:**
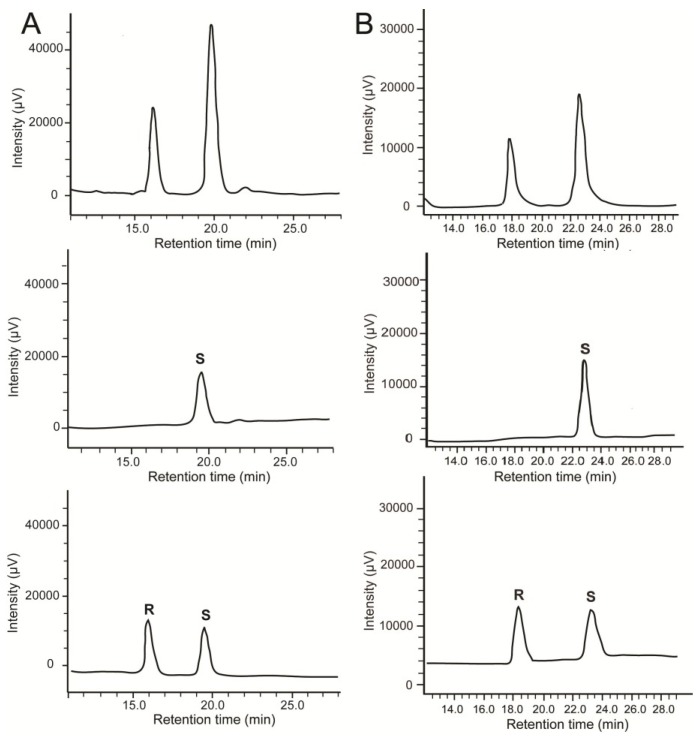
Chiral analysis of (**A**) 15-HEPE and (**B**) 17-HDoHE from *L. danicus* and *L. hargravesii*. Commercial racemic mixtures (bottom), natural compounds (middle) and co-elution of oxylipins with the corresponding racemic mixtures (top).

In order to corroborate the lipoxygenase specificity and complement the elucidation of the biosynthetic pathways, hydroxyeicosapentaenoic acids (HEPEs) and hydroxydocosahexaenoic acids (HDoHEs) of the different species of diatoms were purified on Reverse Phase-HPLC (RP-HPLC) and re-injected on chiral HPLC. The absolute configuration of each metabolite was determined by co-elution with racemic mixture and pure enantiomers. By these means, absolute stereochemistry of 15-HEPE and 17-HDoHE of *L. danicus* and *L. hargravesii* was established as *S* (Figure 4). In *L. convexus*, 18-HEPE and 20-HDoHE gave a single peak that co-eluted with the delayed enantiomers of the commercial mixture of (±)18-HEPE and (±)20-HDoHE, respectively. As *S* enantiomers of HEPEs and HDoHEs are usually eluted later than the corresponding *R* isomers under the chromatographic conditions used for our analysis, these data suggest that LOX metabolism of *L. convexus* specifically produces 18*S*-HEPE and 20*S*-HDoHE. In *L. aporus*, the configuration of 14-HEPE remained unassigned due to the absence of reference compounds. However, this product gave a single chromatographic peak on chiral HPLC. All the hydroxyacids were almost optically pure (enantiomeric excess ~99%), which confirms the pivotal role of enzymatic oxidation in the biosynthesis of NVOs in diatoms.

### 2.3. Quantitative Analysis of Oxylipins

**Figure 5:**
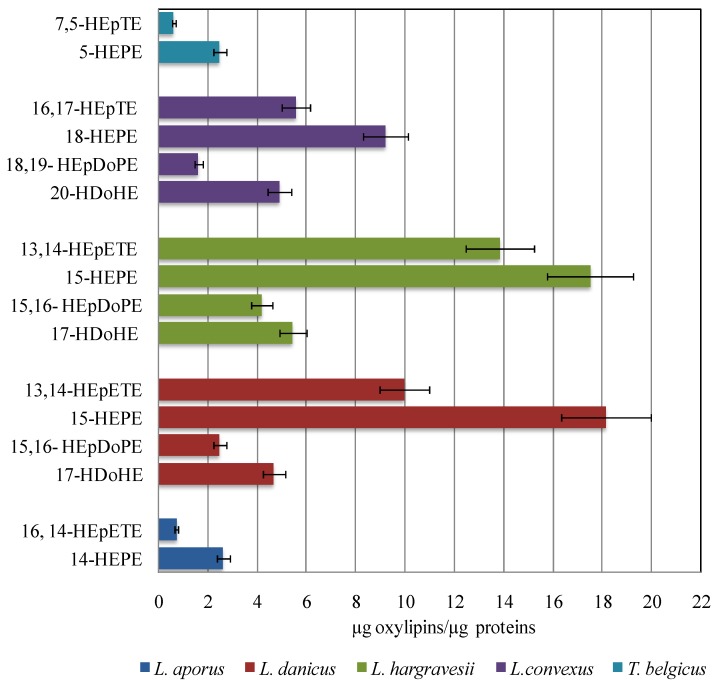
Oxylipin content of *L. aporus*, *L. convexus*, *L. danicus*, *L. hargravesii* and *T. belgicus*. The values are the mean of three replicates. Oxylipin quantification was obtained by LC-MS using 16-hydroxyhexadecanoic acid as internal standard. The data were normalized for protein content (µg oxylipin µg^−1^ protein).

Chemical analysis indicates that hydroxy and epoxyalcohol derivatives of EPA and DHA are the major NVOs in the *Leptocylindrus* genus. Regardless of the positional specificity of the LOX pathways, hydroxyacids were always more abundant than the corresponding epoxy-alcohols, while levels of EPA derivatives were generally higher than the DHA metabolites (Figure 5).

The absolute quantity of major oxylipins (expressed as µg µg^−1^ protein) was comparable in *L. danicus* and *L. hargravesii* (35.5 ± 2.8 and 41 ± 3.2 µg µg^−1^ protein) but slightly lower in *L. convexus* (21.3 ± 1.7 µg µg^−1^ protein). *Leptocylindrus aporus* and *T. belgicus* showed the lowest levels among the species under investigation in this study.

### 2.4. Oxylipins Data Support the DNA-Based Species Categorization and Functionality

Oxylipins analyzed in this study for *Leptocylindrus* species and *Tenuicylindrus belgicus* indicate that the hydroxyacids and epoxyalcohol of EPA are common traits of these two genera. Additionally, DHA derivatives have been distinctly found in the *Leptocylindraceae* species, although in *L. aporus* and *T. belgicus* DHA metabolism was comparatively low. The synthesis of DHA derivatives as the main difference between Leptocylindraceae and other diatoms suggests this as a potential autapomorphic character of this family. However, considering that the family Leptocylindraceae forms the basal clade of the diatom phylogeny [16], the relatively high amount of DHA and presence of DHA derivatives might be an ancestral character, lost in the course of evolution, although we cannot exclude that other genera or species so far not investigated will reveal similar biochemical properties.

**Figure 6:**
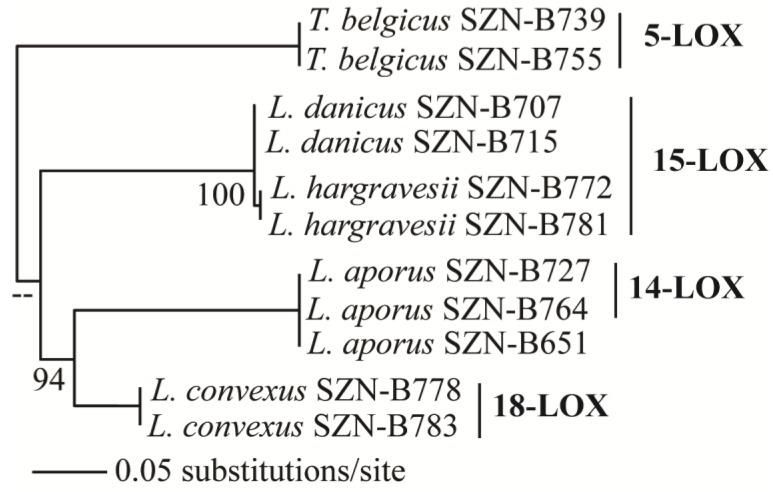
Maximum likelihood (ML) relationships of *Leptocylindraceae* species inferred from SSU rDNA and their respective LOX pathways.

At the genus level, LOX positional specificity of the analyzed strains suggests at least four chemotypes in Leptocylindraceae, corresponding to 14-LOX in *L. aporus*, 18-LOX in *L. convexus*, 15-LOX in *L. danicus* and *L. hargravesii*, and 5-LOX in *T. belgicus* (Figure 3). Notably, these chemotypes correspond to the clades observed with the SSU-rDNA-based phylogeny (Figure 6). In this phylogeny *L. hargravesii* is close to *L. danicus*, and, indeed, the two species have the same LOX position and main oxylipin pattern. However, these are two distinct species, differing for more variable phylogenetic markers such as *rbc*L, *psb*C, and ITS regions as well as for some morphometric characters [16]. In fact there are peaks in the oxylipin profiles, which might correspond to distinctive oxylipins produced in lower amounts, which need further exploration (Supplementary Information, Figure S2). On the other hand, the same LOX and main derivatives might be due to either the more recent separation of the two species or, alternatively to a highly conserved function of the compounds. Similar distribution of LOX activities and products was also noticed in the genus *Pseudo-nitzschia*, for which metabolite analysis indicates that distinct oxylipin patterns can be indicative of even small genetic differences [26]. Apart from *L. danicus* and *L. hargravesii*, LOX type and its derivatives in Leptocylindraceae clearly corroborate DNA-based species discrimination, thus, linking the phylogenetic diversity with functional differences.

While the view that two distinct but phylogenetically related species, *L. danicus* and *L. hargravesii*, may share common biosynthetic pathways points at a conserved functionality, the evidence of several cases of plasticity and intraspecific diversity in the LOX contrasts it. One of the strains of *L. aporus* (SZN-B651) showed no evident LOX activity at all, while the two strains of *L. convexus* differed for the presence of compound with *m/z* 353 (Supplementary Information, Figure S2), which could be the reflection of intraspecific genetic diversity. Similarly in *Skeletonema marinoi*, Gerecht *et al.* [38] observed significant qualitative and quantitative differences in oxylipin production among strains isolated in different time periods from the same place. This result was also consistent with minor genetic differences (LSU) among those strains, suggesting that the diversity of oxylipins can be traced back to intraspecific genetic differences. In addition, notable plasticity is reported in the oxylipin composition and/or relative abundance along the growth curve of diatoms, for instance in *P. pseudodelicatissima* [33] and in *S. marinoi* [39]. In *L. danicus*, as well, there was a positive effect of temperature on the quantity of oxylipin produced, although the composition did not vary [40]. While plasticity in terms of quantitative differences does not interfere with the use of these molecules as a functional support to species delimitation, the observed qualitative differences in some of the species so far analyzed challenge their applicability as markers for species identification.

**Figure 7:**
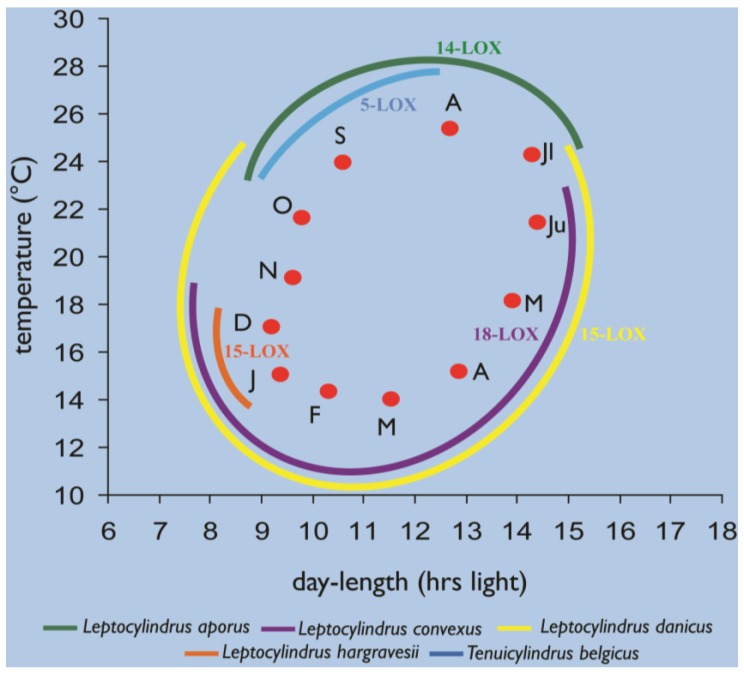
Seasonality of the different *Leptocylindraceae* species at the LTER-MC station, established through combined microscopic observations and strain isolation, with their respective LOX type.

While matching the phylogenetic diversity, the remarkable diversity for oxylipin patterns contrasts the considerable morphological stasis of the genera and species in the *Leptocylindraceae*, all of which are characterized by cylindrical frustules with scarce and rather homogeneous ornamentation, as compared to other diatom families. On the other hand, these species differ in terms of chloroplast number and shapes, which points at possible differences in their physiological response to the light environment. In addition, the seasonal occurrence, duration, and peak intensity also differ among *Leptocylindraceae* species in the GoN, which indicate that they are quite distinct in terms of ecophysiological characteristics, given the marked differences in at least light and temperature conditions over the annual cycle (Figure 7, Supplementary Information, Table S2).

As compared to other morphological characters widely used in identification, *i.e.*, the frustule ultrastructure, oxylipin profiles appear to better reflect these ecophysiological differences, which confirms their role as possible indicators of the functional diversity of diatom species. Of the several roles attributed to these active compounds, including chemical defense against grazing, intercellular communication, and allelochemical interactions [41,42], which are those played by single substances, under which conditions and with which effects on the species performances, should be the object of future investigations.

## 3. Experimental Section

### 3.1. Cultures

Eleven strains of each of the five *Leptocylindraceae* species (*Leptocylindrus aporus*, *L. danicus*, *L. convexus*, *L. hargravesii*, and *Tenuicylindrus belgicus*) were used in this study (Supplementary Information, Table S1). The growth cycle of the individual species was investigated to determine the time needed to reach the stationary phase (see Supplementary Information, Figure S1), which is deemed to correspond to the maximum oxylipin production [33]. To this end, 200 mL flat polystyrene bottles containing 100 mL of K culture medium [43] were inoculated with 50 × 10^3^ cells from a culture in the exponential phase and were allowed to grow under a 12:12 h L:D photoperiod with an irradiance of about 100 µmol photons m^−2^s^−1^ at 20 °C until the end of stationary phase. Cell densities were estimated by counting cells, every day, in a Sedgwick-Rafter chamber.

To obtain the material for the oxylipin analysis, cells were grown into two liter spherical, flat bottomed, glass flasks with one liter of K media, in the same conditions described above. At the end of the stationary phase, the cultures were centrifuged and the culture pellets were collected and frozen until the analysis. In triplicate for each strain, pellets from 300 mL culture were employed in oxylipin analysis and 100 mL in protein quantity estimation.

### 3.2. Oxylipins Analysis

Culture pellets collected for oxylipin analysis were extracted and analyzed according to the methods described in literature [27]. Briefly, cells were suspended in 1 mL of filtered sea-water 0.22 µm, sonicated for 1 min and incubated at room temperature. After 20 min, 1 mL of acetone and 5 µg 16-hydroxy-hexadecanoic acid (16-OH, internal standard) were added. The sample was sonicated again for 1 min and centrifuged at 2000× *g* for 5 min at 5 °C. The supernatant was transferred to a new centrifuge tube and the remaining pellet was resuspended in 2 mL of H_2_O/acetone (1:1, v/v), sonicated for 1 min and centrifuged at 2000× *g* for 5 min. The supernatants were combined and extracted twice with equal volume of CH_2_Cl_2_. The organic layers were combined, dried over dry Na_2_SO_4_, then filtered and evaporated at reduced pressure. The dry extract was methylated with ethereal diazomethane for 30 min, and evaporated again under nitrogen flow.

Methylated extracts were dissolved in methanol to a final concentration of 1 µg µL^−1^ and directly analyzed by LC-MS. The mass spectrometry (MS) method was based on a micro-Quadrupole time-of-flight (micromass Q-TOF micro™, Waters, Milan, Italy) instrument equipped with an electrospray ionization (ESI) source in positive ion mode and a UV photodiode array (PDA 2996 Waters, Milan, Italy) detector (scan range 205–400 nm) for a dual monitoring of the chromatographic runs. For ESI-QToF-MS/MS experiments, argon was used as collision gas at a pressure of 22 mbar. Chromatographic analysis was carried out on a reverse-phase high pressure liquid chromatography (RP-HPLC) using a linear MeOH/H_2_O gradient 75/25 to 100/0 in 30 min with a column flow of 1 mL min^−1^. One tenth of the column flow was channeled by a post-column split to the ESI+ (Q-Tof) MS analyzer and the remaining 9/10 to the UV PDA detector [27].

### 3.3. Protein Content

Protein content was determined by the Lowry method (RC DC protein assay; Bio-Rad, Milan, Italy), according to manufacturer’s instruction. Triton-X100 25% was utilized as cellular detergent. The standard curve was made using bovine serum albumin (BSA, Sigma-Aldrich, Milan, Italy) and a UV/Vis spectrophotometer DU-730 (Beckman Coulter, Fullerton, CA, USA). Quantification was achieved by interpolation from calibration curves at 655 nm.

### 3.4. Chiral Analysis of HEPEs and HDoHEs

All methylated extracts (0.5 mg) were fractionated on reverse-phase high pressure liquid chromatography (RP-HPLC) (Kromasil C-18, 4.6 × 250 mm, Efka Chemicals, Malmoe , Sweden) and the products of interest (14-HEPE, 15-HEPE, 18-HEPE, 17-HDoHE, 20-HDoHE) were collected by UV and MS detection by using a split flow ratio of 1:300 (MS/UV) with a gradient from MeOH:H_2_O 70:30 to 80:20 for 30 min, followed by isocratic elution in 80:20, flow rate 1 mL min^−1^. Each purified compound was then analyzed by chiral phase HPLC using a Chiralcel OD-H (Chiral Technologies Europe, Illkirch-Graffenstaden, France) (4.6 × 250 mm, flow rate 1.5 mL min^−1^, UV detection at 236 nm) using hexane-isopropanol (98:2, v:v) at a flow rate of 1 mL min^−1^ [25]. Each compound was injected alone, and in co-elution with racemic mixture of the corresponding enantiomers. The stereochemistry of each compound was ascertained by co-elution with the corresponding commercial racemic mixtures. (±) 15-HEPE, 15(*S*)-HEPE, (±) 18-HEPE, (±) 17-HDoHE, 17(*S*)-HDoHE, (±) 20-HDoHE were purchased at Cayman Chemical Company (Ann Arbor, MI, USA).

### 3.5. Phylogenetic Analysis

Small subunit ribosomal DNA (SSU rDNA) sequences obtained in previous study were downloaded for the strains used in this study and aligned with Clustal-W. Phylogenetic analysis was done using PAUP, version 4.0b10 [44], and ML tree was constructed using the obtained alignment, and Modeltest version 3.06 [45] was used to select optimal base substitution model. An ML analysis was performed under full heuristic search option in PAUP and the parameters were constrained with values obtained by Modeltest. Bootstrap values associated to internodes were obtained based on 1000 bootstrap replicates.

## 4. Conclusions

The two known Leptocylindraceae genera, *Leptocylindrus* and *Tenuicylindrus*, are both characterized by oxylipin pathways requiring EPA as substrate. In analogy with previous reports on other diatoms, the enantiomeric excess of the corresponding HEPEs confirms the enzymatic control over the oxidative pathway with a preferential addition of oxygen to generate *S* enantiomers. In fact, the chemical data clearly indicate that *L. danicus* and *L. hargravesii* possess 15*S*-LOX enzyme, whereas *T. belgicus* is featured by a 5-LOX. Both activities are rather common in diatoms. On the other hand, *L.* c*onvexus* shows NVOs that are likely derived by 18*S*-LOX, while *L. aporus* is associated to a yet undetermined 14-LOX. As Leptocylindraceae constitute a basal clade in the diatom phylogeny, the EPA pathway might be ubiquitously present in marine diatoms unless the character has been lost over time. In addition, three out of four analyzed *Leptocylindrus* species, as an autapomorphic or as a symplesiomorphic character, produce comparatively high amounts of DHA and oxidize them into its derivatives. According to well-established model of LOX specificity [46], the presence of homolog oxylipins in these species suggests that single enzymes are responsible for parallel metabolism of EPA and DHA. Thus, in analogy with synthesis of 17*S*-hydroperoxide of DHA by arachidonate-dependent 15-LOX in mammals [46,47], it can be proposed that 17*S*-oxylipins of DHA are produced by 15*S* eicosapentanoate:LOX in *L. danicus* and *L. hargravesii*. To the best of our knowledge this is the first report of high levels of DHA enzymatic derivatives in diatoms and in marine organisms, although these compounds might have been overlooked so far because of their trace presence. As DHA derived oxylipins could reflect the DHA concentration in the diatom cells, further studies are necessary to address the distribution of these oxylipins in other diatom species.

In the current study, we also report a good match between taxonomic classification of individual *Leptocylindraceae* species and distribution of LOX activities and NVO patterns. Oxylipin-based discrimination of species indeed might provide a fertile interface to diatom morphological and molecular species identification, as well as a functional validation of the different taxa pointing at possible eco-physiological differences among these species.

In this view, the finding of DHA derivatives adds new compounds to the known diatom oxylipins diversity, increasing the number of traits useful for the functional discrimination. Even for species-rich diatom genera, individual compounds or unique combinations of different compounds can reflect the species identity. On the other hand, the process of oxylipin synthesis is dynamic and is influenced by the intraspecific-genetic diversity and abiotic factors. Therefore, prior to its application as biomarker for functional diversity analysis, it is important to decipher the factors that trigger their synthesis and the physiological conditions that modulate the process. Thus, greater efforts in characterizing the pathways of oxylipin synthesis in diverse diatoms species and under different conditions not only can help understanding its applicability as a potential functional diversity biomarker, but can also shed light on numerous other processes of ecological relevance in which these molecules are involved.

## Supplementary Files

Supplementary File 1 Supplementary Information (PDF, 474 KB)
