# Supplementary material for: Oxylipin Diversity in the Diatom Family Leptocylindraceae Reveals DHA Derivatives in Marine Diatoms
Source: Mar Drugs. 2014 Jan 17;12(1):368–84. doi: 10.3390/md12010368 (PMC3917278; doi:10.3390/md12010368)
Supplement: Supplementary File 1 — Supplementary Information (PDF, 474 KB) [file marinedrugs-12-00368-s001.pdf]

Supplementary Information

Table S1. List of strains used in the study, their date of isolation and SSU rDNA GenBank accession numbers.

Table S2. Seasonality of *Leptocylindraceae* species with their bloom time and LOX type.

Figure S1. Typical growth curves established by cell density for *Leptocylindrus* species and *T. belgicus* (*n* = 3). Cell densities were estimated by counting cell number every day, using a Sedgwick-Rafter counting chamber.

Figure S2. LC-MS chromatograms of (**A**) *L. aporus B724*; (**B**) *L. convexus* B783; (**C**) *L. convexus* B778; (**D**) *L. danicus* B707; (**E**) *L. hargravesii* B781; (**F**) *T. belgicus* B755.

Table S1. List of strains used in the study, their date of isolation and SSU rDNA GenBank accession numbers.

Strain	Date of Isolation	SSU rDNA
Leptocylindrus aporus		
**SZN-B727**	03/08/2010	KC814810
**SZN-B764**	18/11/2010	KC814810
**SZN-B651**	21/08/2010	KC814810
Leptocylindrus convexus		
**SZN-B778**	25/01/2011	KC814811
**SZN-B783**	25/01/2011	KC814811
Leptocylindrus danicus		
**SZN-B707**	15/02/2010	KC814808
**SZN-B715**	15/06/2010	KC814808
Leptocylindrus hargravesii		
**SZN-B772**	21/12/2010	KC814809
**SZN-B781**	25/01/2011	KC814809
Tenuicylindrus belgicus		
**SZN-B739**	02/10/2010	KC814812
**SZN-B755**	19/10/2010	KC894152

Table S2. Seasonality of *Leptocylindraceae* species with their bloom time and LOX type.

Species	Season	Peak Months	Max Cell No. Cells L^{-1}		Oxylipin Pathway	
L. aporus	Jul–Oct	Jul–Aug	$<10^5$–10^6	counted	14-LOX	
L. convexus	Dec–Mar	Jan–Feb	$<10^2$–10^3	guessed	18-LOX	probably mixed with *L. danicus*
L. danicus	Nov–Jul	May–Jun	$<10^4$–10^5	counted	15-LOX	probably mixed with *L. convexus*
L. hargravesii	Dec–Jan	Dec–Jan	$<10^2$	guessed	15-LOX	probably mixed with *L. danicus and L. convexus*
T. belgicus	Aug–Oct	Sept–Oct	$<10^4$–10^5	counted	5-LOX	

Figure S1. Typical growth curves established by cell density for *Leptocylindrus* species and *T. belgicus* (n = 3). Cell densities were estimated by counting cell number every day, using a Sedgwick-Rafter counting chamber. Arrowheads indicate the stage at which cells were harvested for analysis (Stationary phase).

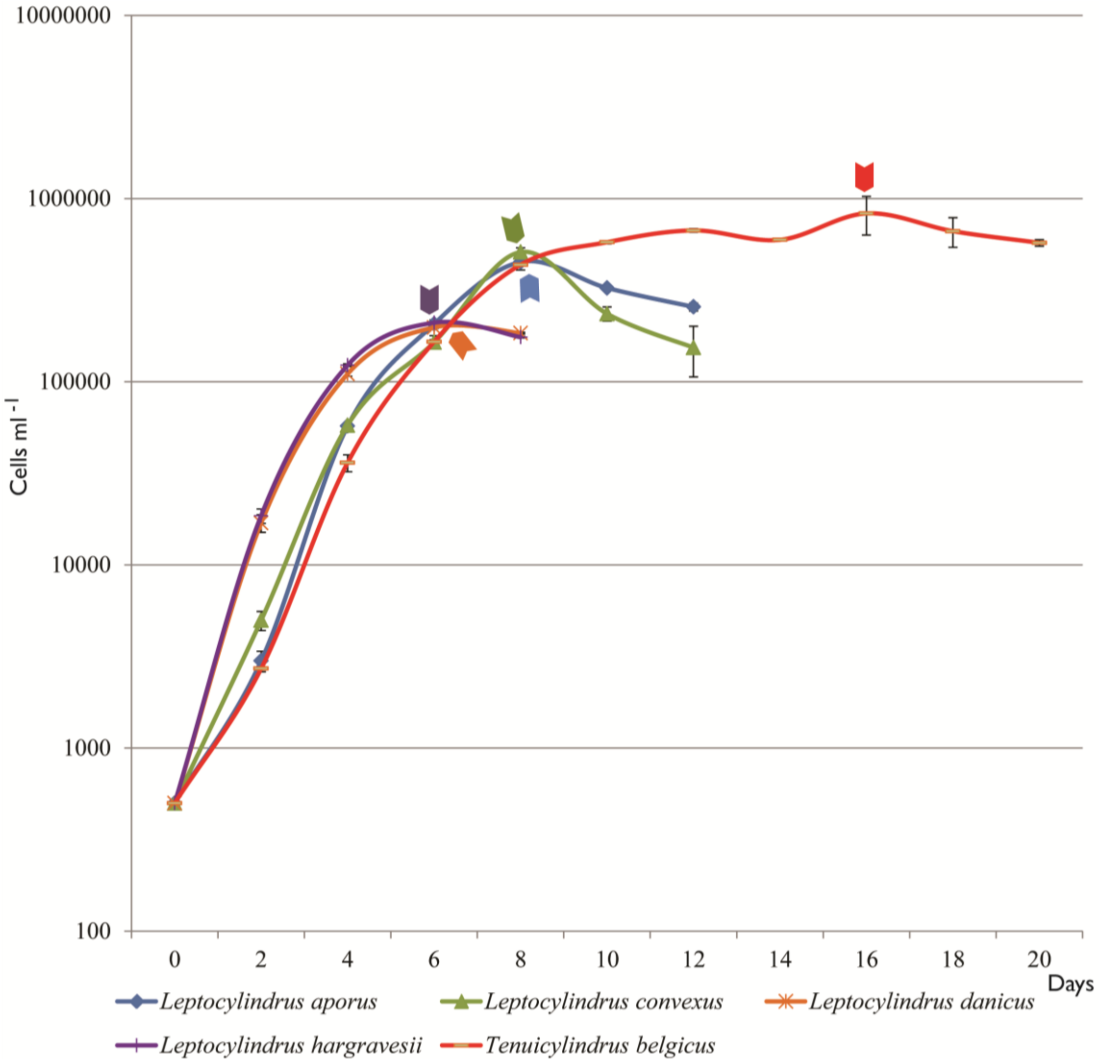

Figure S2. LC-MS chromatograms of (**A**) *L. aporus B724*; (**B**) *L. convexus* B783; (**C**) *L. convexus* B778; (**D**) *L. danicus* B707; (**E**) *L. hargravesii* B781; (**F**) *T. belgicus* B755.

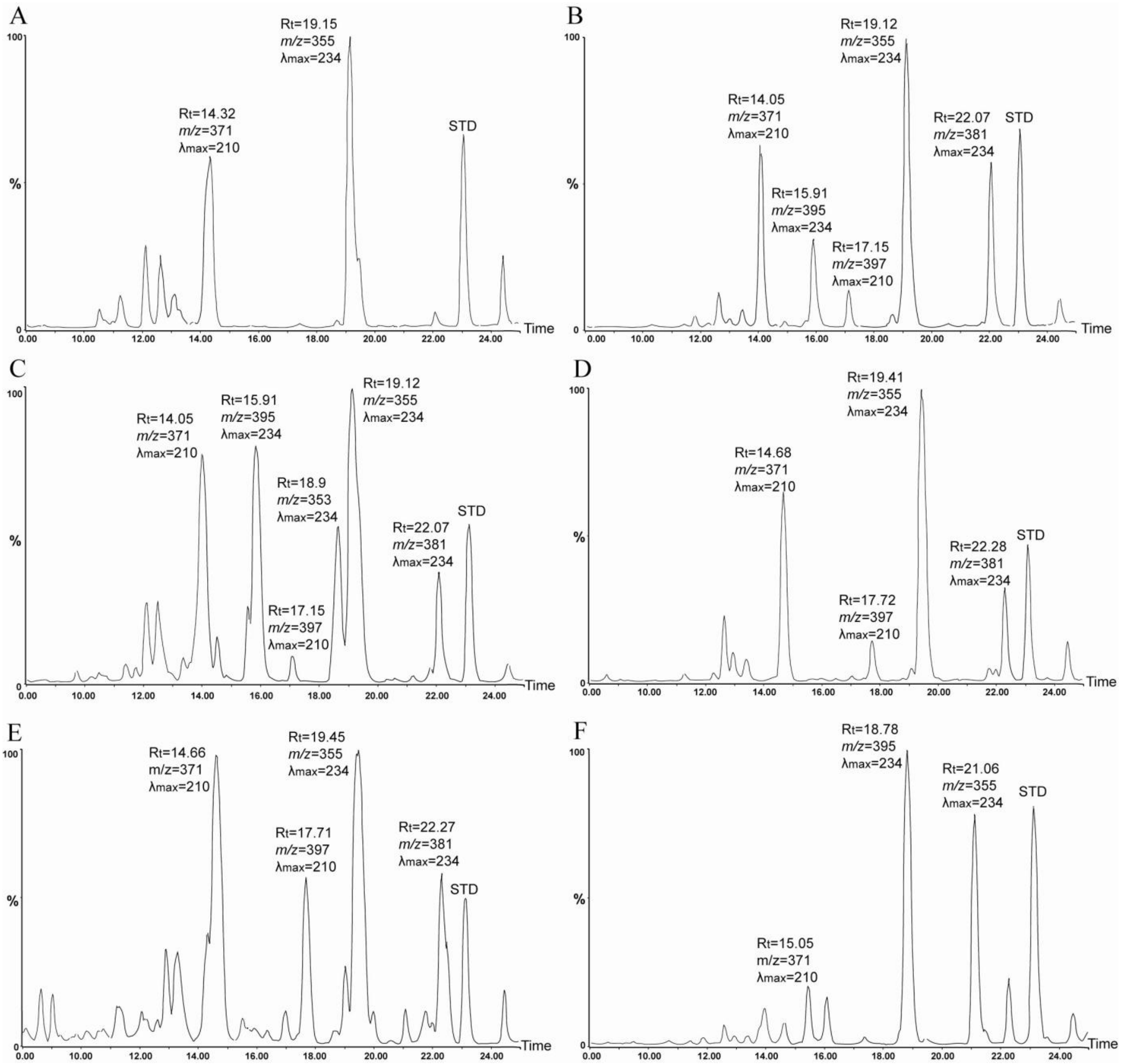

© 2014 by the authors; licensee MDPI, Basel, Switzerland. This article is an open access article distributed under the terms and conditions of the Creative Commons Attribution license (http://creativecommons.org/licenses/by/3.0/).
